# Editorial: Advances in bioactive peptides and functional properties of foods

**DOI:** 10.3389/fchem.2024.1538944

**Published:** 2025-01-03

**Authors:** Brij Pal Singh, Pedro Ferreira Santos, Silvia Luna-Suárez, Cristian Jimenez Martínez

**Affiliations:** ^1^ University Centre for Research and Development (UCRD), Chandigarh University, Mohali, Punjab, India; ^2^ Department of Chemical Engineering, Faculty of Science, University of Vigo (Campus Ourense), Vigo, Spain; ^3^Instituto de Agroecoloxía e Alimentación (IAA), Campus Auga, University of Vigo, Vigo, Spain; ^4^ Instituto Politécnico Nacional, Centro de Investigación en Biotecnología Aplicada, Tepetitla, Mexico; ^5^ Instituto Politécnico Nacional, Escuela Nacional de Ciencias Biológicas, México, Mexico

**Keywords:** bioactive peptide, functional food, health benefits, encapsulation, packaging films

Recent research on bioactive peptides (BAPs) has provided interesting insights into their production, characterization, and impact on health. The BAPs display a variety of biological functions like antioxidant, antihypertensive, anti-diabetic, antimicrobial, immunomodulatory, and anticancer effects. These actions are observed when BAPs are released from their original proteins by enzymatic hydrolysis, fermentation, or during food processing (Singh et al., 2022). In this Research Topic, we focus on the recent advancements in functional foods and production, characterization, and *in silico* methods for obtaining BAPs from a range of biological resources.

In this Research Topic, we focus on a study investigating the production, evaluation, and molecular docking of BAPs by fermenting sheep milk with an indigenous strain of *Lactobacillus fermentum* (Pipaliya et al.). The inhibitory activities of BAPs on different targeted enzymes such as angiotensin-converting enzyme (ACE), lipase, α-glucosidase, and α-amylase were investigated. These different enzymes were selected because they have certain influences on the human body system. ACE helps regulate blood pressure; lipase was targeted for hyperlipidemia treatment, and α-glucosidase and α-amylase are involved in diabetes (Singh et al., 2022). In general, the authors observed that fermented sheep milk (FSM) promotes more than 60% inhibition of the activity of the tested enzymes. Initially, the growth characteristics of the *L. fermentum* strain were optimized in sheep milk, and maximum proteolytic activity was found after 48-h incubation. Further characterization of BAPs using bioinformatic tools corroborated the potential antihypertensive and antidiabetic properties.

Encapsulation is a well-known strategy to enhance the stability, shelf life, bioaccessibility, and bioavailability of bioactives (Aguilar-Toalá et al., 2022). In an updated review, the authors explored the effect of encapsulation on the stability and biological activity of plant-derived BAPs. Plant-based proteins and BAPs have recently gained attention for their potential in both health-promoting properties and serving as a sustainable source (Singh et al., 2023a). Recent evidence suggests that the consumption of plant-based proteins reduces blood pressure and cholesterol levels, acts against inflammation and cancer, and promotes immune system modulation. Interestingly, the peptides released upon plant protein hydrolysis may be the prime component responsible for the above-mentioned health effects. However, plant-derived BAPs are generally found to be less stable physicochemically and are hydrolyzed quickly prior to reaching the target site; therefore, their application in functional foods and nutraceuticals is restricted (Karami and Akbari-Adergani, 2019). In this context, encapsulation may be the best way to conserve the stability and biofunctionality of peptides. The stability and bioavailability of encapsulated peptides are enhanced by achieving small, spherical particles with a narrow size distribution. Sodium alginate is one of the most suitable wall materials for shielding peptides from extrinsic factors such as light, temperature, pH, and enzymatic activity. Yet the peptide’s bioaccessibility is reduced due to the formidable molecular structure created by alginate, which may hinder its release and absorption. However, this issue can be resolved by incorporating lower quantities of different gums or mucilage into the mixture of wall materials (Pérez-Pérez et al.).

In the past 10 years, there has been an increase in studies focusing on developing active packaging films using biopolymers and natural bioactive compounds. These active films help increase the shelf life of foods by inhibiting microbial growth. Additionally, these packaging films can decompose naturally, which benefits the circular bioeconomy and makes them environmentally friendly (Singh et al., 2023b). In this Research Topic, an original work that has explored chia seed-derived nanocomposite films for food packaging applications is described (Morales-Olán et al.). Nevertheless, research indicates that chia-based films exhibit inadequate mechanical characteristics; thus, combining natural polymers with nanoparticles may be an effective approach to enhance film properties. Furthermore, nanoparticles can trap bioactive substances and produce an active film. The purpose of the research was to assess how chia flour, glycerol, and chia extract-loaded chitosan nanoparticles affect the physical, mechanical, barrier, structural, and antioxidant characteristics of chia-based nanocomposite films. It was noticed that as the amount of chia flour in the films increased, the water vapor permeability decreased, but the tensile strength and antioxidant capacity improved. Moreover, the incorporation of chitosan nanoparticles resulted in the formation of diverse films, but the nanoparticles had a major impact on the thickness and enhanced both the mechanical and antioxidant characteristics of the films (Morales-Olán et al.).

Fermented foods have a rich history of use and are known to enhance consumer health as they contain beneficial microbes, primarily lactic acid bacteria (LAB). Another intriguing study within this Research Topic investigated the feasibility and functional influence of probiotic LAB in salami, a fermented meat product (Mani-López et al.). They have used two strains of lactic acid bacteria *i.e., Lactiplantibacillus plantarum* and *Lactobacillus acidophilus* as a starter and probiotic microorganism, respectively. The findings indicate that *L. acidophilus*, *L. plantarum*, or a combination of both can serve as a starter culture for fermentation of meat. Nevertheless, utilizing *L. acidophilus*, either alone or in combination, is a feasible choice that maintains the qualities of the fermented food and could enhance the advantages of probiotic supplements. It is also observed that the kind of bacteria utilized did not have an effect on the final product’s sensory characteristics (Mani-López et al.).

Yogurt, a widely consumed fermented milk product worldwide, is often fortified with various food components, like fruit pulp, to enhance its taste and nutritional value. Another study in this Research Topic delved into a functional food: synbiotic mango fruit-infused yogurt and also analyzed the biofunctional attributes of the BAPs extracted from it. Most of the time, mixtures of BAPs with varying molecular weights have been shown to be more effective than individual peptides alone. Likewise, this research revealed that the BAPs in the 10-kDa fractions exhibited stronger antioxidant and ACE-inhibitory properties compared to smaller fractions (Minj and Vij).

Overall, this research highlights the importance of alternative BAPs and functional food innovation in addressing global health challenges and promoting sustainable practices. Future research should focus on optimizing production processes, exploring new bioresources, and developing scalable solutions that combine health benefits with ecological responsibility.
